# Case report and literature review of transthyretin amyloidosis with p.Ser43Asn mutation presenting in China

**DOI:** 10.3389/fcvm.2025.1625485

**Published:** 2025-09-02

**Authors:** Kebinuer Abulikemu, Lei Hong, Qinghua Zhong

**Affiliations:** Department of Cardiovascular Medicine, Shenzhen Longgang Central Hospital, Shenzhen, Guangdong, China

**Keywords:** p.Ser43Asn mutation, familial transthyretin amyloidosis, cardiac amyloidosis, amaurosis fugax, genetic testing

## Abstract

**Background:**

Familial transthyretin amyloidosis (ATTR) is a rare hereditary disorder marked by abnormal protein fibril deposits that impair multiple organ functions. Although over 130 mutations have been identified, the p.Ser43Asn variant, which has been associated with amyloidosis involving thyroxine-binding globulin (TBG), is scarcely reported in the literature, especially within the Chinese population.

**Case presentation:**

We report a case of a 50-year-old male presenting with recurrent episodes of amaurosis fugax. The patient had a family history of cardiac disease. Auxiliary examinations revealed myocardial hypertrophy, and the electrocardiogram indicated conduction delays and abnormal QRS complexes. Nuclear imaging suggested cardiac amyloidosis, and cardiac MRI indicated non-ischemic cardiomyopathy. Genetic testing identified a c.128G > A (p.Ser43Asn) mutation, and pathological examination confirmed ATTR amyloidosis. Both pathological findings and mass spectrometry analysis supported this diagnosis.

**Discussion:**

The p.Ser43Asn variant likely impacts TTR protein dimerization and stability, leading to protein misfolding and amyloid fibril deposition in tissues. Early diagnosis is crucial due to the significant heterogeneity of this condition. Our findings emphasize the importance of increased diagnostic awareness, multidisciplinary collaboration, and regional genetic screening. Comparisons with similar cases reported in the literature highlight the shared clinical presentations and diagnostic challenges.

**Conclusion:**

This case adds valuable insight into the clinical manifestations and diagnostic challenges of ATTR amyloidosis with the p.Ser43Asn mutation, particularly in the Chinese population. Future research should aim to develop effective treatment strategies and explore the genetic and environmental factors influencing the phenotypic variability of this rare disorder.

## Introduction

Amyloidosis is a rare disease characterized by the accumulation of abnormal protein fibrils forming amyloid deposits, resulting in the functional impairment of multiple organs and tissues (1). Transthyretin-derived amyloidosis (ATTR) is a prominent hereditary form of systemic amyloidosis, characterized by significant genetic heterogeneity worldwide (2, 3). ATTR can present with cardiac or nervous system involvement or as a systemic disease. Although TTR contributes minimally to thyroxine transport (accounting for ∼15% of serum binding), its destabilizing mutations drive amyloidogenesis and cause profound clinical manifestations unrelated to thyroid dysfunction, as evidenced by the safety profile of TTR-silencing therapies. However, p.Ser43Asn mutation and familial amyloidosis are still scarcely reported in the literature.

In this case report, we present a patient with the p.Ser43Asn mutation associated with ATTR, which involves amyloid deposits related to TBG. We also provide a comprehensive review of the current literature to highlight and contextualize this rare condition.

## Case presentation

The patient is a 50-year-old male admitted to the neurology department in March 2023 due to recurrent episodes of amaurosis fugax. He had no history of hypertension, diabetes, or gastrointestinal diseases. Family history ncluded the mother's heart disease and two brothers diagnosed with “hypertrophic cardiomyopathy”, treated inadequately for heart failure, and both died from cardiac causes before age 55.

Physical examination showed clear consciousness and slightly yellowish skin. The patient's vital signs were within normal limits: blood pressure was 120/80 mmHg, heart rate was 75 beats per minute (regular), respiratory rate was 16 breaths per minute, body temperature was 36.6°C, and oxygen saturation was 97% on room air. Neurological examination revealed that the patient was alert, oriented, and had fluent speech. Pupillary reflexes were normal bilaterally, with no facial asymmetry or signs of facial palsy. Motor coordination was intact, with normal gait and no signs of ataxia. Sensory examination showed no abnormalities in light touch, pain, or vibration sensation in the extremities. Muscle strength was normal (5/5) in all four limbs, with no weakness or atrophy. Deep tendon reflexes, including knee and ankle reflexes, were normal bilaterally, and the Babinski sign was negative.

Visual acuity testing demonstrated normal vision in both eyes (right eye: 1.0, left eye: 1.0). Visual field testing revealed no significant deficits, and fundoscopic examination showed no abnormalities in the retina, with normal light reflexes and no signs of retinal hemorrhage or edema.

No third heart sound or peripheral edema were noted. A systolic murmur was detected at the left lower sternal border, which increased with Valsalva maneuver, consistent with hypertrophic cardiomyopathy.

**Figure 1:**
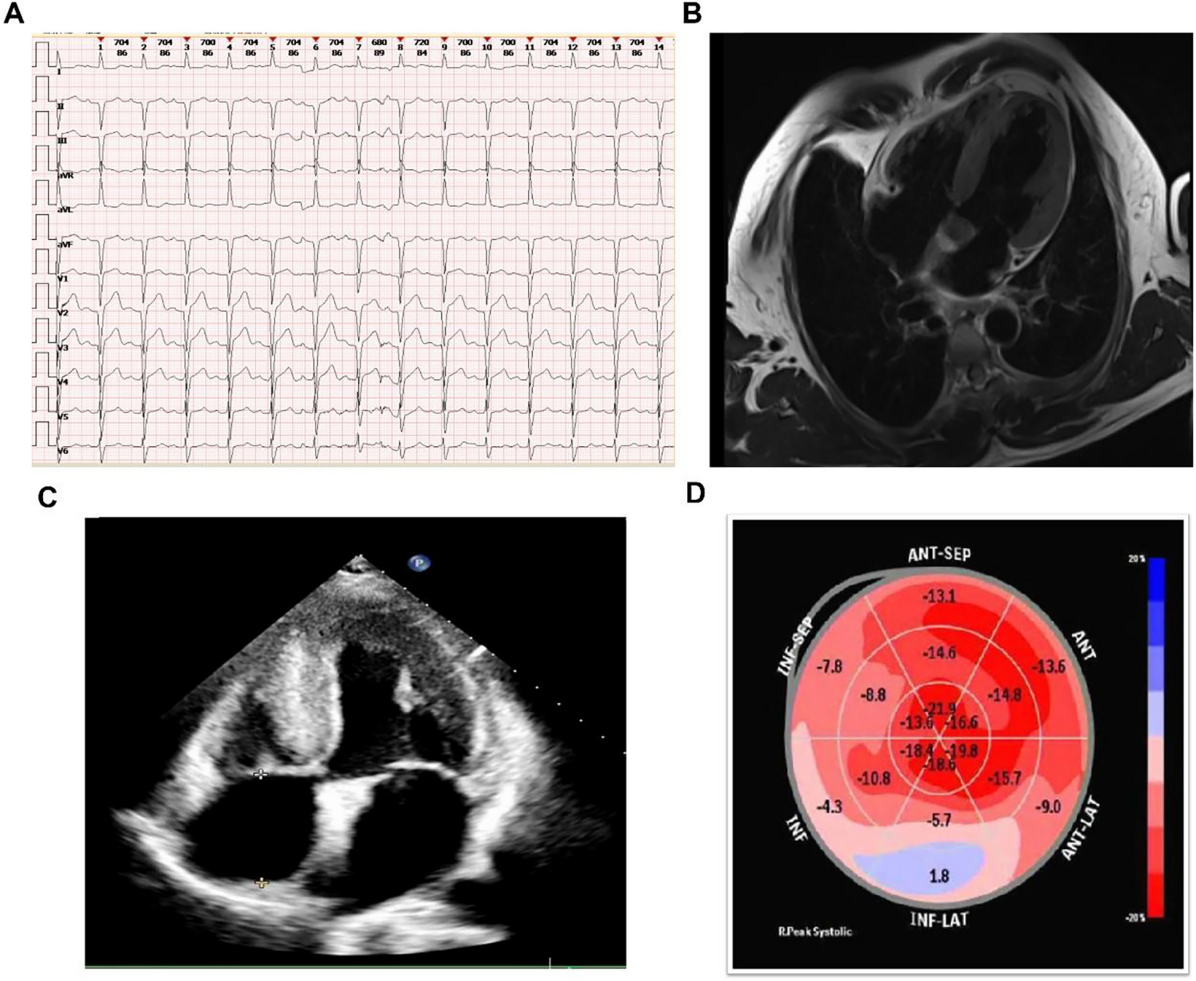
Diagnostic imaging results. **(A)** ECG showing intraventricular conduction delay, widened QRS complexes, abnormal R wave progression, and low voltage. **(B)** Cardiac MRI showing diffuse left ventricular thickening and delayed enhancement. **(C)** Echocardiography showing myocardial hypertrophy. **(D)** Strain analysis from echocardiography showing significant reduction in myocardial movement across multiple segments.

**Figure 2:**
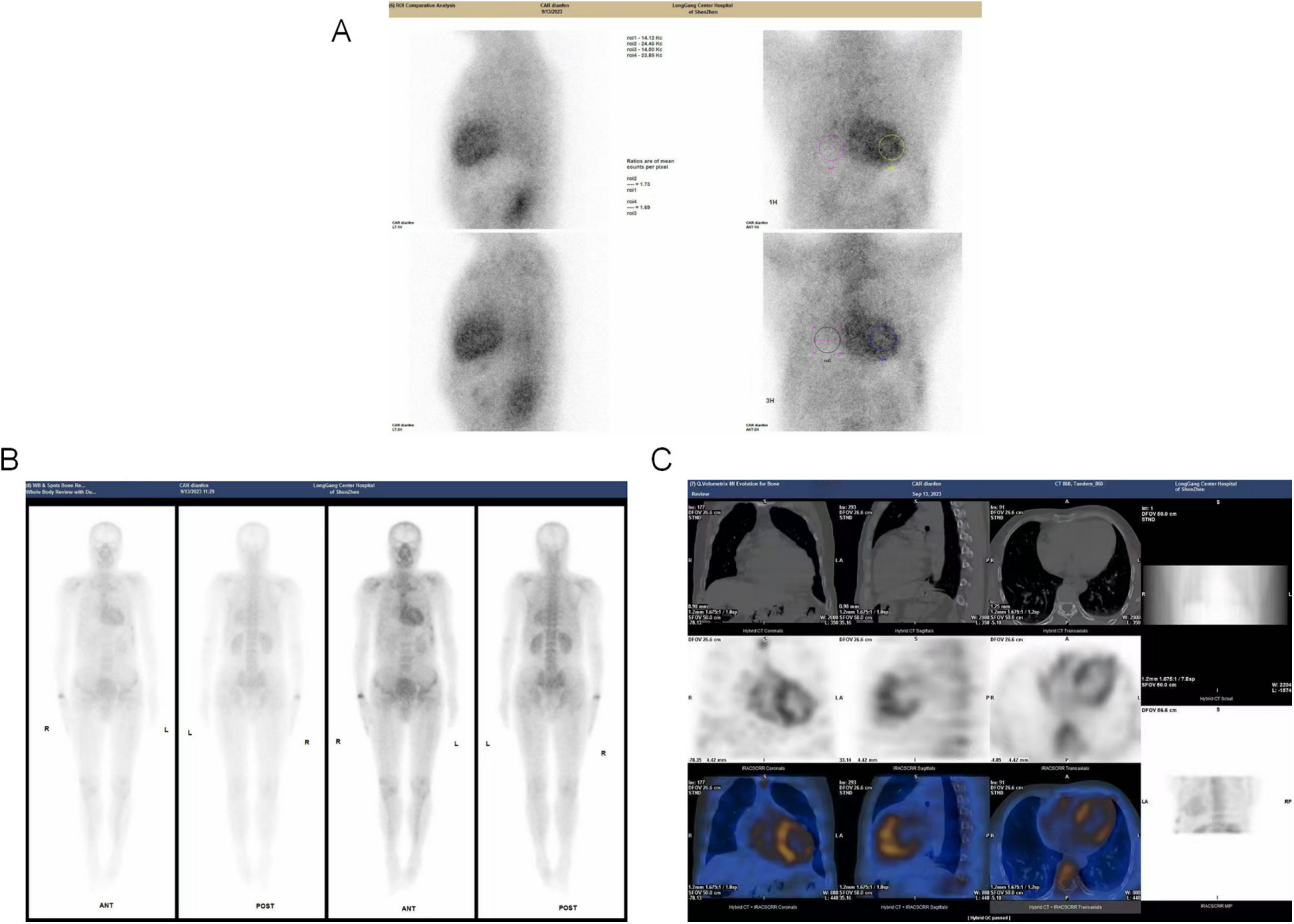
Multimodal ^99m^Tc-PYP scintigraphy and SPECT/CT imaging. **(A)**: Planar anterior and left lateral chest images acquired 1 h after intravenous injection of 20 mCi ^99m^Tc-PYP. Myocardial uptake is diffusely increased and significantly higher than adjacent ribs and sternum, consistent with Perugini Grade 3. Image quality and contrast are satisfactory. **(B)** Whole-body planar scintigraphy and ROI analysis obtained at 3 h post-injection. The myocardium continues to exhibit intense tracer retention, exceeding adjacent osseous structures (visual score remains Grade 3). Region-of-interest (ROI) analysis shows a heart-to-contralateral-lung (H/CL) ratio of 1.73 at 1 h and 1.69 at 3 h. Additional focal uptake is observed in the right wrist and anterior segments of the left 7th and 8th ribs. Tracer distribution in kidneys and bladder is physiological. **(C)** SPECT/CT fusion images of the chest at 3 h post-injection. Images reveal clear tracer accumulation within the left ventricular myocardium, markedly exceeding radiotracer concentration in adjacent ribs and sternum. Mild retention is noted in the blood pool. CT component shows irregular cortical outline at the left 7th and 8th anterior ribs.

Auxiliary examinations, including routine blood tests, urinalysis, complete biochemistry, thyroid function tests, immunofixation electrophoresis, and serum free light chain (FLC) analysis. These tests revealed a normal kappa/lambda ratio of 1.23 (reference range: 0.26–1.65) and the absence of monoclonal bands. Testing for anti-nuclear antibodies was negative, results of auxiliary examinations were unremarkable. The electrocardiogram (as shown in the Figure 1A) indicated intraventricular conduction delay with widened, atypical QRS complexes, abnormal R wave progression, and low voltage in some leads. Cardiac enzymes (troponin I: 0.044 ng/ml; normal range: <0.04 ng/ml) and NT-proBNP (1,260 pg/ml; normal range: <125 pg/ml) were elevated, indicating more pronounced cardiac strain or dysfunction. Coronary CTA excluded coronary artery disease. Brain MRI and EEG were unremarkable. Echocardiography indicated myocardial hypertrophy (Figure 1C), the strain analysis results indicate a significant reduction in myocardial movement across multiple segments (Figure 1D). Cardiac MRI revealed diffuse thickening of the left ventricular myocardium and delayed enhancement of the basal segments of the left ventricular wall and endocardium, indicating non-ischemic cardiomyopathy and potential amyloid cardiomyopathy (Figure 1B). After intravenous administration of 20 mCi ⁹⁹^m^Tc-PYP, planar imaging was performed with the patient in the supine position. At 1 h post-injection, anterior and left lateral chest planar images were acquired, showing diffusely increased myocardial uptake significantly greater than that of the adjacent ribs and sternum (Perugini Grade 3), with satisfactory image quality and contrast (Figure 2A). At 3 h post-injection, additional whole-body planar imaging and repeat anterior/lateral chest views were obtained. Myocardial tracer retention remained markedly elevated, again exceeding uptake in surrounding osseous structures (visual score Grade 3). ROI-based semiquantitative analysis revealed a heart-to-contralateral-lung (H/CL) ratio of 1.73 at 1 h and 1.69 at 3 h, supporting the diagnosis of ATTR cardiac amyloidosis (Figure 2B). SPECT/CT fusion imaging of the chest performed at the 3 h mark demonstrated intense focal tracer accumulation in the left ventricular walls, exceeding that of adjacent ribs and sternum, along with mild blood pool activity. CT revealed irregular cortical outlines of the left 7th and 8th anterior ribs (Figure 2C).

While current ESC guidelines (4) permit the non-invasive diagnosis of ATTR cardiac amyloidosis in patients with positive bone scintigraphy (Perugini grade 2–3) and absence of monoclonal protein, an endomyocardial biopsy was performed in this case for two specific reasons: (1) To exclude concomitant light-chain (AL) amyloidosis or other rare amyloid subtypes, given the atypical presentation with isolated amaurosis fugax and absence of neuropathy; (2) To provide definitive histopathological characterization for research purposes, including mass spectrometry-based proteomic subtyping, given the rarity of the p.Ser43Asn variant in Asian populations. This approach aligns with guideline recommendations for equivocal cases or research settings.

**Figure 3:**
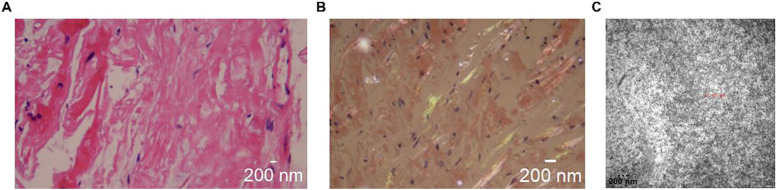
Pathological examination results. **(A)**: H&E staining showing homogeneous, red-staining material deposited between muscle fibers. **(B)** Congo red staining with apple-green birefringence under polarized light. **(C)** Ultrastructural pathology under electron microscopy howing rigid, non-branching fibrous deposits in myocardial stroma (8–12 nm).

**Figure 4:**
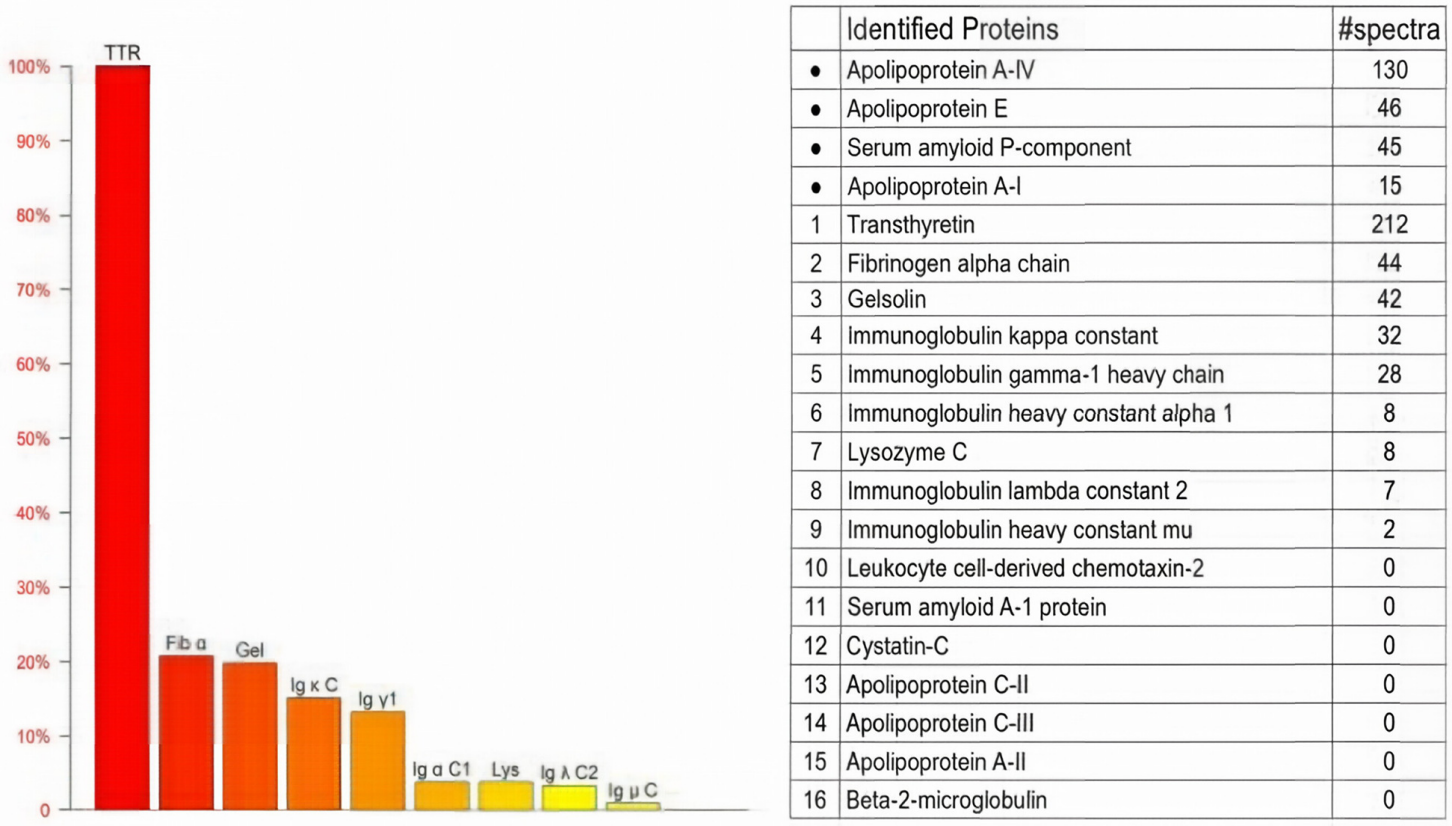
Mass spectrometry analysis. Mass spectrometry showing high abundance of ApoAIV, ApoE, SAP, and TTR, indicating ATTR amyloidosis.

Genetic testing revealed a c.128G > A (p.Ser43Asn) mutation (Table 1). In the pathological examination, we observed the following results. To further confirm the diagnosis and exclude alternative amyloid subtypes, an endomyocardial biopsy was performed. Hematoxylin and Eosin (H&E) staining (Figure 3A) revealed homogeneous, red-staining, structureless material deposited between muscle fibers and bundles, while special staining (Figure 3B) demonstrated Congo red positivity with apple-green birefringence under polarized light, consistent with amyloid deposition. Ultrastructural pathology (Figure 3C) demonstrated abundant deposits of fibrous material in the myocardial stroma, approximately 8–12 nm in diameter, exhibiting a rigid, non-branching, and disorganized pattern. Mass spectrometry analysis (Figure 4) identified a high abundance of amyloid chaperone proteins ApoAIV, ApoE, and SAP in the pathological sample, indicating amyloid deposition. Among the known typing proteins, TTR had the highest relative abundance, suggesting the ATTR type.

**Table 1:** Genetic testing results.

Category	Details
Gene	*TTR*
Location	chr18:29172917
Transcript	NM_000371.4; exon 2
Nucleotide/Amino Acid	c.128G > A/p.Ser43Asn
Homozygous/Heterozygous	Heterozygous
Frequency in Normal Population	—
ACMG Pathogenicity Analysis (Score)	Pathogenic
Disease/Phenotype (Inheritance Pattern)	1. Familial transthyretin amyloidosis (AD) 2. Hyperthyroxinemia (AD) 3. Carpal Tunnel Syndrome Type 1 (AD)
Variant Source	—

The patient was initiated on diflunisal (250 mg twice daily) as bridging therapy under close monitoring, considering the following contextual factors: (1) Regional Drug Accessibility: At the time of treatment initiation (March 2023), guideline-recommended TTR stabilizers (tafamidis) and RNAi therapies (patisiran/inotersen) were not commercially available or reimbursed in our healthcare system; (2) Evidence for Stabilization: Diflunisal exhibits TTR-stabilizing effects demonstrated in clinical trials (5), with NT-proBNP reduction (1,260→890 pg/ml) and symptom improvement observed in this case; (3) Transition Plan: The patient is currently undergoing evaluation for transition to tafamidis following its recent local approval, with serial cardiac biomarkers guiding escalation. Regular follow-up assessments at 3-month intervals include serial electrocardiograms, serum NT-proBNP monitoring to evaluate cardiac function. The patient's NT-proBNP levels have decreased from 1260 pg/ml at baseline to 890 pg/ml at 6-month follow-up, accompanied by subjective improvement in exertional dyspnea.

## Discussion

### Clinical interpretation

This report presents a rare variant (p.Ser43Asn) of ATTR characterized by the clinical manifestation of amaurosis fugax. Amaurosis fugax can be caused by numerous clinical conditions, with neurological disorders and arrhythmias often being considered as primary causes. In this particular case, echocardiographic evidence of myocardial hypertrophy might typically lead to a diagnosis of hypertrophic cardiomyopathy, often halting further investigation. However, syncope can be an overlooked clinical manifestation of amyloidosis, potentially linked to involvement of the nervous system and autonomic dysfunction. This underscores the critical need for heightened awareness and comprehensive diagnostic evaluations in such presentations.

ATTR variant amyloidosis is a predominant type of familial amyloidosis characterized by diverse pathogenic genes and clinical phenotypes. The p.Ser43Asn variant leads to a single amino acid substitution, potentially affecting the dimerization and stability of the TTR protein. This instability can result in misfolding and aggregation, forming amyloid fibrils that deposit in tissues and disrupt normal functioning. Understanding the clinical characteristics of ATTR is crucial, as more than 130 mutations have been identified, with Val30Met and Val122Ile being the most prevalent (6). TTR functions in the transport and regulation of thyroid hormones in the body. ATTR is characterized by slowly progressive peripheral sensory-motor neuropathy, autonomic neuropathy, non-neuropathic cardiomyopathy, nephropathy, vitreous opacities, and central nervous system amyloidosis (7). Symptom onset generally occurs between the ages of 30 and 50 years, with early-onset diseases often presenting with autonomic neuropathy, including orthostatic hypotension, alternating constipation and diarrhea, nausea and vomiting, delayed gastric emptying, impotence, anhidrosis, urinary retention, and urinary incontinence. Despite the importance of understanding TTR mutations, reports on familial amyloidosis associated with these mutations, particularly p.Ser43Asn, are relatively scarce. Therefore, this case is significant in enhancing our understanding of the clinical characteristics of ATTR amyloidosis.

### Literature comparison

The study by Diana Elizabeth Luzuriaga Carpio et al. presents three cases of ATTR with the rare p.Ser43Asn variant in Ecuador (8). These cases illustrate diverse clinical manifestations, diagnostic challenges, and management issues similar to those in our case report. In Case 1, a 59-year-old male experienced syncope, breathing difficulty, general weakness, and limb edema, initially misdiagnosed with irritable bowel syndrome, later confirmed to have hypertrophic cardiomyopathy via imaging and genetic testing, eventually succumbing to heart failure due to delayed diagnosis. In Case 2, a 49-year-old male presented with significant limb edema and abdominal symptoms, revealing heart blockages and hypertrophic features, confirmed by genetic testing to have the p.Ser43Asn variant, leading to a referral for cardiac transplantation. In Case 3, a 62-year-old male with complete AV block and hypertrophic cardiomyopathy received a pacemaker but ultimately died from disease complications, with genetic testing also revealing the p.Ser43Asn variant. These cases share similarities with our reports, particularly in clinical presentation, disease progression, and diagnostic hurdles, with recurrent symptoms such as myocardial hypertrophy, heart failure, frequent syncope, and multi-system involvement. Genetic testing confirmed the p.Ser43Asn variant in all cases, reinforcing the familial and hereditary nature of the condition. Important clinical and management insights highlight the crucial need for awareness and molecular diagnostic capabilities to prevent adverse outcomes, emphasize multi-disciplinary approaches and regular monitoring to mitigate systemic damage and fatal outcomes, and suggest regional genetic screening programs to manage clustered occurrences effectively. A recent study identified patients with the Ser23Asn mutation in the Chinese population, highlighting the importance of genetic screening and endomyocardial biopsy for diagnosing hereditary transthyretin cardiac amyloidosis. Similar to our case, these patients exhibited significant cardiac involvement, including myocardial hypertrophy and heart failure (9). This finding along with our comparison to Luzuriaga Carpio et al. (8), underscore the need for comprehensive genetic evaluation and early intervention to improve outcomes, emphasizing the importance of recognizing diverse clinical presentations and genetic variants associated with ATTR, particularly across different populations. We recommend regional genetic screening and interdisciplinary collaboration to enhance awareness, facilitate timely diagnosis, and implement comprehensive care strategies, ultimately supporting patients with this rare disease.

In this case, the transient amaurosis fugax may be mechanistically linked to dynamic hemodynamic fluctuations. Although cerebrovascular imaging was unremarkable, echocardiography revealed asymmetric septal hypertrophy and a systolic murmur accentuated by Valsalva maneuver, suggesting intermittent dynamic left ventricular outflow tract (LVOT) obstruction. While atypical for ATTR, this phenomenon has been documented in amyloidosis with significant septal thickening, where amyloid infiltration alters ventricular compliance and mitral apparatus kinematics, mimicking hypertrophic cardiomyopathy (10). Crucially, Bartolini et al. demonstrated that 36% of ATTR patients exhibit abnormal blood pressure responses during cardiopulmonary exercise testing, including exertional hypotension (11). Such hemodynamic instability could precipitate transient cerebral hypoperfusion during postural changes or exertion, potentially explaining amaurosis fugax without structural cerebrovascular lesions. Additionally, stress-induced myocardial perfusion-contraction mismatch, as reported in AL amyloidosis by Ong et al. (12), may further contribute to cardiac output fluctuations in ATTR. Although exercise stress echocardiography—a valuable tool for unmasking latent LVOT gradients (13) was contraindicated here due to conduction abnormalities, the integrated evidence supports dynamic obstruction and autonomic dysregulation as plausible contributors to transient visual loss.

### Future directions

Once the disease is detected, it progresses rapidly and has a high mortality rate. Some case reports have discussed treatment options for cardiac amyloidosis caused by the p.Ser43Asn mutation, primarily involving the installation of cardiac assist devices or Heart-liver transplantation. A patient diagnosed with rare p.Ser43Asn variant, which required the implantation of a pacemaker, exhibited rapid deterioration in his condition following the initial diagnosis. This led to the implantation of a left ventricular assist device and eventually necessitated a heart transplant (14). Subsequently, Mueller et al. reported a case involving a 46-year-old female of German-Italian descent carrying the same variant. She initially presented with restrictive cardiomyopathy and severe heart failure without evidence of polyneuropathy or other organ involvement. Unfortunately, she succumbed to septic complications while awaiting a heart transplant. Her family history did not reveal other members diagnosed with amyloidosis or cardiac disease, including close relatives on her mother's side, though there was no information available regarding her Italian father and this ancestry (15).

Another case by Daoko et al. identified the same variant in a 41-year-old male from Peru who presented with angina and shortness of breath. This patient had a family history suggestive of hypertrophic cardiomyopathy (HCM) leading to sudden cardiac death (his mother and two brothers in Peru). His amyloidosis was confined to the heart without involvement of other organs. The patient underwent the implantation of an implantable cardioverter-defibrillator and was subsequently referred for liver and heart transplantation (16).

In a different case, Castaño et al. described a 41-year-old male from Ecuador, of Italian and Spanish descent, who exhibited a mixed phenotype of cardiac and neurological involvement (14). This was the first report of the variant being associated with neurological symptoms. A seven-generation pedigree was constructed, indicating 29 family members with cardiac-related deaths, 24 of whom died before the age of 55. The patient successfully underwent a heart and liver transplant. His liver was used in a domino transplant for a 77-year-old patient with end-stage liver disease secondary to nonalcoholic steatohepatitis. One year post-transplant, the recipient developed cardiac amyloidosis, which alongside previously reported cases, highlights the exceedingly rare and rapidly progressive nature of this disease (17).

The hallmark of cardiac amyloidosis is progressive cardiomyopathy. In this report, the patient's syncope led to the discovery of ATTR amyloidosis, with cardiac MRI, nuclear imaging, and myocardial biopsy indicating primary cardiac involvement. The patient is currently undergoing treatment with diflunisal, which has been proven effective in treating ATTR. Nonetheless, the treatment options for this rare variant remain limited, underscoring the need for further research to develop more effective therapeutic strategies and interventions.

These case reports and literature reviews provide substantial insights into the various clinical manifestations, treatments, and outcomes associated with ATTR amyloidosis, particularly focusing on the p.Ser43Asn variant. Further research and accumulated data will be essential in improving the understanding and management of this rare disorder.

In conclusion, the p.Ser43Asn variant of ATTR presents with diverse and challenging clinical manifestations. Our case, alongside similar reports, underscores the necessity for heightened clinical awareness, timely molecular diagnostics, and multidisciplinary management approaches. Future research should focus on developing more effective therapeutic strategies and understanding the genetic and environmental factors contributing to the phenotypic variability of this rare disorder.

## Data Availability

The original contributions presented in the study are included in the article/Supplementary Material, further inquiries can be directed to the corresponding author.

## References

[1] Iakovleva I Hall M Oelker M Sandblad L Anan I Sauer-Eriksson AE. Structural basis for transthyretin amyloid formation in vitreous body of the eye. Nat Commun. (2021) 12(1):7141. 10.1038/s41467-021-27481-4 34880242 PMC8654999

[2] Adams D Gonzalez-Duarte A O’Riordan WD Yang C-C Ueda M Kristen AV Patisiran, an RNAi therapeutic, for hereditary transthyretin amyloidosis. N Engl J Med. (2018) 379(1):11–21. 10.1056/NEJMoa1716153 29972753

[3] Benson MD Waddington-Cruz M Berk JL Polydefkis M Dyck PJ Wang AK Inotersen treatment for patients with hereditary transthyretin amyloidosis. N Engl J Med. (2018) 379(1):22–31. 10.1056/NEJMoa1716793 29972757 PMC12611561

[4] Garcia-Pavia P Rapezzi C Adler Y Arad M Basso C Brucato A Diagnosis and treatment of cardiac amyloidosis: a position statement of the ESC working group on myocardial and pericardial diseases. Eur Heart J. (2021) 42(16):1554–68. 10.1093/eurheartj/ehab072 33825853 PMC8060056

[5] Berk JL Suhr OB Obici L Sekijima Y Zeldenrust SR Yamashita T Repurposing diflunisal for familial amyloid polyneuropathy: a randomized clinical trial. JAMA. (2013) 310(24):2658–67. 10.1001/jama.2013.283815 24368466 PMC4139164

[6] Maurer MS Hanna M Grogan M Dispenzieri A Witteles R Drachman B Genotype and phenotype of transthyretin cardiac amyloidosis: THAOS (transthyretin amyloid outcome survey). J Am Coll Cardiol. (2016) 68(2):161–72. 10.1016/j.jacc.2016.03.596 27386769 PMC4940135

[7] Tozza S Severi D Spina E Di Paolantonio A Iovino A Guglielmino V A compound score to screen patients with hereditary transthyretin amyloidosis. J Neurol. (2022) 269(8):4281–7. 10.1007/s00415-022-11056-4 35279758 PMC9293821

[8] Luzuriaga Carpio DE Abrigo Maldonado BR Villacorta H. Experience of hereditary amyloidosis with rare variant in ecuador: case reports. Med Sci. (2024) 12(4):58. 10.3390/medsci12040058 PMC11503339 39449414

[9] Yu T-P Hou J Yang T-J Chen X-Q Chen Y-C. Hereditary transthyretin cardiac amyloidosis proven by endomyocardial biopsy: a single-centre retrospective study and literature review. Acta Cardiol. (2024) 79(4):436–43. 10.1080/00015385.2023.2257521 37768132

[10] Mookadam F Haley JH Olson LJ Cikes M Mookadam M. Dynamic left ventricular outflow tract obstruction in senile cardiac amyloidosis. Eur J Echocardiogr. (2006) 7(6):465–8. 10.1016/j.euje.2005.09.002 16236554

[11] Bartolini S Baldasseroni S Fattirolli F Silverii MV Piccioli L Perfetto F Poor right ventricular function is associated with impaired exercise capacity and ventilatory efficiency in transthyretin cardiac amyloid patients. Intern Emerg Med. (2020) 16:653–60. 10.1007/s11739-020-02474-1 32918156

[12] Ong KC Wells Askew J Dispenzieri A Maleszewski JJ Klarich KW Anavekar NS Abnormal stress echocardiography findings in cardiac amyloidosis. Amyloid. (2016) 23(2):124–31. 10.1080/13506129.2016.1176020 27132767

[13] Salinger T Hu K Liu D Herrmann S Lorenz K Ertl G Cardiac amyloidosis mimicking severe aortic valve stenosis—a case report demonstrating diagnostic pitfalls and role of dobutamine stress echocardiography. BMC Cardiovasc Disord. (2017) 17(1):86. 10.1186/s12872-017-0519-0 28330445 PMC5361717

[14] Castaño A Bokhari S Brannagan TH Wynn J Maurer MS. Technetium pyrophosphate myocardial uptake and peripheral neuropathy in a rare variant of familial transthyretin (TTR) amyloidosis (Ser23Asn): a case report and literature review. Amyloid. (2012) 19(1):41–6. 10.3109/13506129.2011.638682 22149423 PMC4934899

[15] Maurer MS Schwartz JH Gundapaneni B Elliott PM Merlini G Waddington-Cruz M Tafamidis treatment for patients with transthyretin amyloid cardiomyopathy. N Engl J Med. (2018) 379(11):1007–16. 10.1056/NEJMoa1805689 30145929

[16] Daoko J Elnahar Y Kersh KE Mohammad N Shamoon F. Cardiac MRI detection of a rare case of familial cardiac amyloidosis (Ser23Asn): case report with literature review. Rep Med Imaging. (2010):123–7.

[17] Dixit N Castano A Farr MJ Traub R Lentzsch S Brown RS Rapidly progressive transthyretin-mediated amyloidosis in a domino liver transplant recipient of a Ser23Asn donor. J Clin Neuromuscul Dis. (2016) 17(3):142–5. 10.1097/CND.0000000000000110 26905915

